# Supplementary material for: Resolving the full spectrum of human genome variation using Linked-Reads
Source: Genome Res. 2019 Apr;29(4):635–45. doi: 10.1101/gr.234443.118 (PMC6442396; doi:10.1101/gr.234443.118)
Supplement: Supplemental Material [file supp_gr.234443.118_Supplemental_longranger-2.2.zip › longranger-2.2.tar.bz2!/lr2.2-public/lib/stan/cmdstan/stan/doc/api/html/classstan_1_1optimization_1_1_model_adaptor.html]

Stan: stan::optimization::ModelAdaptor< M > Class Template Reference


|  |  |
| --- | --- |
|  | Stan  2.5.0  probability, sampling & optimization |


- Main Page
- Related Pages
- Namespaces
- Classes
- Files

- Class List
- Class Index
- Class Hierarchy
- Class Members

All Classes Namespaces Files Functions Variables Typedefs Enumerations Enumerator Friends Macros Pages

- stan
- optimization
- ModelAdaptor

Public Member Functions |
List of all members

stan::optimization::ModelAdaptor< M > Class Template Reference

`#include <bfgs.hpp>`

|  |  |
| --- | --- |
| Public Member Functions | |
|  | ModelAdaptor (M &model, const std::vector< int > &params\_i, std::ostream \*msgs) |
|  | |
| size\_t | fevals () const |
|  | |
| int | operator() (const Eigen::Matrix< double, Eigen::Dynamic, 1 > &x, double &f) |
|  | |
| int | operator() (const Eigen::Matrix< double, Eigen::Dynamic, 1 > &x, double &f, Eigen::Matrix< double, Eigen::Dynamic, 1 > &g) |
|  | |
| int | df (const Eigen::Matrix< double, Eigen::Dynamic, 1 > &x, Eigen::Matrix< double, Eigen::Dynamic, 1 > &g) |
|  | |

## Detailed Description

### template<class M> class stan::optimization::ModelAdaptor< M >

Definition at line 297 of file bfgs.hpp.

## Constructor & Destructor Documentation

template<class M >

|  |  |  |  |
| --- | --- | --- | --- |
| stan::optimization::ModelAdaptor< M >::ModelAdaptor | ( | M & | *model*, |
|  |  | const std::vector< int > & | *params\_i*, |
|  |  | std::ostream \* | *msgs* |
|  | ) |  |  |

inline

Definition at line 306 of file bfgs.hpp.

## Member Function Documentation

template<class M >

|  |  |  |  |
| --- | --- | --- | --- |
| int stan::optimization::ModelAdaptor< M >::df | ( | const Eigen::Matrix< double, Eigen::Dynamic, 1 > & | *x*, |
|  |  | Eigen::Matrix< double, Eigen::Dynamic, 1 > & | *g* |
|  | ) |  |  |

inline

Definition at line 385 of file bfgs.hpp.

template<class M >

|  |  |  |  |  |
| --- | --- | --- | --- | --- |
| size\_t stan::optimization::ModelAdaptor< M >::fevals | ( |  | ) | const |

inline

Definition at line 311 of file bfgs.hpp.

template<class M >

|  |  |  |  |
| --- | --- | --- | --- |
| int stan::optimization::ModelAdaptor< M >::operator() | ( | const Eigen::Matrix< double, Eigen::Dynamic, 1 > & | *x*, |
|  |  | double & | *f* |
|  | ) |  |  |

inline

Definition at line 312 of file bfgs.hpp.

template<class M >

|  |  |  |  |
| --- | --- | --- | --- |
| int stan::optimization::ModelAdaptor< M >::operator() | ( | const Eigen::Matrix< double, Eigen::Dynamic, 1 > & | *x*, |
|  |  | double & | *f*, |
|  |  | Eigen::Matrix< double, Eigen::Dynamic, 1 > & | *g* |
|  | ) |  |  |

inline

Definition at line 341 of file bfgs.hpp.

---

The documentation for this class was generated from the following file:

- src/stan/optimization/bfgs.hpp


---

[ Stan Home Page ]

*© 2011–2014,
Stan Development Team.*
